# The Important Element in the Treatment of Cases of Prostatic Obstruction

**Published:** 1918-02

**Authors:** Arthur L. Chute

**Affiliations:** Boston, Mass.


### BUFFALO MEDICAL JOURNAL

Yearly Volume 73 FEBRUARY, 1918 Number 7

### ORIGINAL ARTICLES

The right is reserved to decline papers not dealing with practical medical and surgical subjects, and such as might offend or fail to interest readers. Contributors are solely responsible for opinions, methods of expression and revision of proof.

### The Important Element in the Treatment of Cases of Prostatic Obstruction.

DR. ARTHUR L. CHUTE, Boston.

The subject which I bring before you is one that has been of great interest to me for several years past and to the clinical consideration of which I have devoted a good deal of time. Much of it deals with rather vague and not clearly demonstrated points, whose working is more easily shown on a group of patients than described in a paper. Much that relates to it is hard to give in definite terms or figures and any attempts to do this are quite apt to be misleading.

When one considers the amount of trauma done in carrying out a prostatectomy—either suprapubic or perineal—as compared with the much greater degree of injury often done in many of the serious pelvic operations, he is struck with the much greater mortality that attends prostatectomy. It is impossible to get figures that bear very definitely on this point, but I think that most men will agree with me, that in the hands of good general surgeons the mortality from prostatectomy is much greater than the mortality following pelvic operations, even when the latter are attended with greater mutilation and more profound primary shock.

It may be contended that while these operations are analogous in the region in which they are performed, they are wholly unlike in that the pelvic operations are carried out on patients who are much younger than those upon whom prostatectomy is done; also that with the pelvic cases there is a more accurate haemostasis. I think one must admit that the element of age is in favor of the patients upon whom the pelvic operations are done; also that it is true that in most instances there is a more definite haemostasis in the pelvic cases; but even when all these points are taken into consideration, I believe there is still a disproportionate mortality and also a much stormier convalescence after prostatectomy than these elements satisfactorily account for. Besides this, there is a treacherousness about prostatectomy that is rarely seen in the other condition. Some of the prostatectomy cases, who are apparently the best risks, die of vague and more or less ill defined conditions, sometimes considered hemorrhage, though the bleeding is no greater than that seen in cases apparently similar, that make a good recovery: sometimes the death is considered due to shock, though these patients are often in fair condition immediately following operation and the shock develops only slowly, death following in from three to five days after operation.

All these things would seem to point to the probability that the mortality in these prostatic operations depends upon some factor other than the trauma produced by operation. Formerly it was held that different types of operative technique would do away with the high mortality, and many were the modifications that were proposed to diminish the shock, control hemorrhage, etc. These modifications improved the results of the individuals who suggested them, but did not prove of great use in the hands of the general public; demonstrating, to my mind, that these modifications were not really of fundamental importance, as regards their life-saving character, but simply that the men who proposed them and who, as a rule, had had a considerable experience with such cases, had unconsciously developed an intuitive knack for making these patients do better; that they did not appreciate upon what this depended and were, therefore, incapable of imparting it to others; that the various refinements of technique had to do with the betterment of the end results in the hands of the general public rather than the improvement of their mortality. In other words, that the cause of the mortality was more deep-lying than the mere operative technique and depended upon some element which we were not accustomed to deal with in general surgical problems.

In looking for the respect in which these patients differed from ordinary general surgical patients, it was found that the element almost invariably present—especially in cases that did badly—was a residual urine, often a very considerable one, and it began to appear as though the important element which we were over-looking in the care of these cases must be related in some way to this condition.

Between five and six years ago, the probability of this was brought home to me forcibly. At this time I had under my care, within a very short period, two patients suffering with prostatic obstruction with over-distended bladders. Their condition was so poor that it was obviously impossible to do an immediate prostatectomy. One, who had a very light-colored, but not infected urine, was catheterized; at first intermittently, later by an in-lying catheter. This procedure was carried out with the greatest care it was possible for me to observe. In spite of every precaution, he became infected, went down hill and died. In the last days of his life, this man developed the dry tongue, hiccough, slightly distended abdomen, nausea, mental dullness, bloody and diminished urine that I had so often seen in fatal cases following prostatectomy. The second patient was a man who had been catheterized for some little time before I saw him. His urine was bloody and foul; he had a temperature and was evidently thoroughly infected. His bladder was opened suprapubically under ether. He died within about twenty-four hours. Although both these patients presented the signs so often seen in fatal post-operative prostatic cases it was perfectly evident they had died, not of prostatectomy, but of kidney infection.

These cases raised in my mind the probability that much of the mortality that we had supposed to be due to some particular risk inherent to the removal of the prostate, must be laid to the renal element that entered into the problem and that this was in some way connected with the presence of a residual urine. Further observation showed that the patients who died or who had stormy convalescences, almost without exception presented a considerable amount of residual urine; that usually the urine was light in specific gravity and low in solids. It was noted that in some of these cases the urine was uninfected. These, at first sight, seemed to be in especially favorable condition, though it was found that they did particularly badly. In others the urine was infected and one would say off-hand that they were less favorable for operative procedure; but further observation demonstrated the surprising fact that the patients with infected bladders were distinctly better risks than the patients who presented non-infected bladders. At this time, I formulated the working hypothesis that the danger in prostatectomy was almost entirely dependent upon the renal function; that the presence of any considerable amount of residual urine seriously interfered with this; that surgical attacks upon the prostate itself were not attended with any especial or peculiar danger, and that the minute one could relieve the kidney of certain baleful effects that often went hand in hand with the prostatic obstruction, he could get the mortality down from a very considerable one to a very small one.

As a proof of the accuracy of this hypothesis,—I was able, applying this principle, to reduce my mortality following prostatectomy from that was considerable to one that was small, averaging in different series about 4% or 5%, this including operations on both benign and malignant prostates, also operations done by both the perineal and suprapubic routes. I have done a series of about forty consecutive cases on one or two occasions, without a death. I do not believe that this is due to chance, but to the fact that the principle used in these cases was the correct one as far as the lessening of mortality is concerned.

In my belief the great element of danger in prostatectomy lies, not in the operation itself, but in the fact that we have to perform these operations upon people whose kidney function, (a very vital process), has been seriously impaired. This impairment is due to back pressure brought about by the presence of residual urine. It occurs under two general types. First, we have the aseptic cases, the ones where the urine is perfectly transparent, of very low gravity, poor in solids. In some of these cases there is a definite dilatation of the ureter with dilatation of the kidney pelvis and backing up of urine on the kidney; in other instances no dilatation of the ureter or kidney is noticeable and the back pressure seems to take place through a normal or pretty nearly normal ureter. Often these patients, due to lack of infection, do not have any considerable amount of frequency or other symptom referred to the bladder. They do have, at times, nocturnal incontinence. The symptoms from which they suffer are apt to be digestive and very frequently they present marked examples of cachexia urinaria—loss in weight, lack of appetite, distended abdomen, nausea and sometimes even vomiting. It may be, in fact, that the only signs which point to the urinary tract are objective ones,—a full bladder, as felt suprapubically, and a very light gravity urine, containing very little in the way of solids; to these may be added at times, as previously mentioned, nocturnal incontinence. It seems as though a condition of this sort may at times be wholly mechanical, depending solely upon back pressure; at least, this would seem probable from the fact that kidneys which, under this influence of back pressure, excrete a very pale, albuminous urine, poor in solids, will often show, months after operation and relief of the back pressure, a normal urine of good gravity, containing a normal amount of solids, thus showing that the kidney is able to recuperate in a way that would seem impossible if tissue changes had taken place in it to any particular extent. I often think of this process as a temporary embarrassment of renal function. The second type is composed of cases in which we have the same element of back pressure as in aseptic cases, but with an infected urine. In these instances, there is usually, besides the element of embarrassment due to back pressure, an element of renal destruction due to the pyelitis or pyelonephritis that has taken place. In one way these cases are less dangerous than the aseptic ones; in another way they may be more so. They have the advantage of having received a certain immunity through infection, which the aseptic cases do not possess. They have the disadvantage that the suppurative process may have destroyed the renal tissue to a variable, perhaps a very considerable extent; or, if it has not actually destroyed this, it may have set in motion a suppuration which will eventually do so. It is among the cases of this type that we see patients whom it is impossible to restore to health, since their renal function is actually destroyed by infection to a degree that prevents anything more than a slight interruption in their fatal progression. In the aseptic cases, we have a condition which is capable to a greater degree of permanent recovery, in that the changes which have produced their renal incompetence are capable of great betterment, if not, indeed, almost of cure. While the possibilities for permanent improvement in these cases are greater, the danger in dealing with them at the time of operation is also much greater for this reason: these patients have, due to their back pressure, dilated pelvis and perhaps tubules, are wholly unprotected from ascending infection and are very susceptible, since they have not received the immunity possesed by the kidneys already slightly infected.

This explains in a few words what I believe to be the great danger that we have to meet in prostatectomy; that is, kidneys whose function has been seriously crippled through back pressure, sometimes with an aseptic urine, more frequently with an infected urine. The relief of back pressure is always attended with a certain amount of risk—in my opinion, with the possibility of very great risk—unless that is carried out in the proper way. Our problem is how to meet the situation with the least danger. The older way of dealing with urinary back pressure was by the use of the catheter, used either intermittently or constantly. This is still useful in certain cases. I believe, however, its use should be almost completely restricted to the cases with infected urines. My reason is that with the normal bacterial flora of the urethra, it is utterly impossible for anyone to catheterize, over any length of time, a man who has an over-distended, atonic bladder, without producing an infection. The problem here is entirely different from the catheterizing of the patient with an acute retention due to injury or to acute disease. It compares only to those instances of repeated catheterization which we see in spinal lesions, where infection invariably takes place, first of the bladder then of the kidneys. When such an infection occurs, the limitation of that infection depends upon elements which no person can foresee or guage; some of the elements that enter into it are the degree of dilatation of the ureter, the resistance of the patient, the virulence of the culture,—all these are elements, but they are things that we can change but little. The way which seems to me to afford the best chance of immunity from infection of the upper urinary tract in these cases of bladders that are over-distended with aseptic urine is the suprapubic opening of the over-distended bladder and the insertion of a large drainage tube, which will allow no puddling of septic urine or no intermittence of drainage and will also afford every opportunity for the dilated ureter and pelvis to contract. In other words, a two-stage operation.

This, of course, often does not offer absolute immunity from ascending renal infection, but adequate drainage, according to the sound surgical practice employed in other locations, should offer a greatly diminished probability of an ascending infection, when compared with the catheter, used either intermittently or continuously. The temporary interruption of the drainage, due to plugging of the catheter's eye or to its slipping, the mechanical urethritis that the catheter so often produces, seem to me to make infection absolutely sure and not to provide the adequate drainage that the other method produces, (adequate drainage being our best method for combating the spread of infection, no matter whether it is situated in the cellular tissues or the urinary tract). The passage of a catheter into the bladder through a trocar introduced above the pubes may be safer than the use of a catheter introduced through the urethra, but it seems to me to have no advantage over the introduction of a large suprapubic tube through an open incision made under the use of local anaesthesia, when one regards the element of shock and sepsis. It certainly has the disadvantage in the way of drainage which a small tube has over a larger one; furthermore, the employment of the larger tube has the advantage that it leaves a walled-off, granulating tract which can be used as an avenue of approach to the prostate at the time of its removal.

My summary of the methods of relieving back pressure would be that the infected cases that are sufficiently tolerant to be easily drained by catheter, should have the bad effect of kidney back pressure counteracted by the use of continuous catheter drainage; that this should be continued till the signs of renal insufficiency, due to back pressure, have disappeared. This will rarely be in less than a week and will often be much longer. Every aseptic case presenting back pressure should have his bladder opened suprapubically under local anaesthesia, a large drainage tube placed in position and left till all signs of intoxication, due to faulty renal action, have disappeared; in other words, that he have a two-stage operation. It is imperative that this cystostomy be done under local anaesthesia, else it loses its whole point;—the use of ether on kidneys that are embarrassed by aseptic back pressure invites disaster.

The question immediately arises, "Is it necessary to do this in all cases presenting urinary back pressure?" One must undoubtedly answer, "It is not." When, however, one is asked in what cases it is safe to omit this procedure, the answer must be guarded. The reason for this lies in the impossibility of estimating two things; the first is our inability to estimate the potential power of the kidneys or, to use a current phrase, the ability of the injured kidney to "come back" after the relief of back pressure. The second is our ignorance of the extent to which the kidneys will be further crippled by the progression of any infection which is already present or which may be introduced at the time of prostatectomy, in such cases as are not infected. This inability to draw definite conclusions from the various functionally tests as regards these two very important points applies to all tests alike. The difficulty is that these tests simply tell us the power of the kidneys as it is at the time that the test is made. When the kidneys are hampered by back pressure the relief of this back pressure will also greatly improve kidney function. None of the functional tests has this power of clairvoyance which we need to tell us what the renal function will be when the kidney has been relieved of this embarassment of back pressure. Furthermore, no matter how ample the margin of safety may be, as indicated by the functional tests, we cannot be sure that we may not need it all, because of the crippling which can come from the diminished renal power that follows the progression of infection introduced at the time of operation, or by the injury to renal function brought about by the irritating action of ether on the kidneys. This makes evident the difficulty and fallacy of accepting any arbitrary figure of kidney efficiency as the criterion as to whether one may safely attempt prostatectomy or not. It may mislead us in one of two ways: it may encourage us to operate when we should not or it may, because of the low figures of the arbitrary scale, prevent us from attempting operations which are perfectly safe. One must make his decision to operate in each case upon the findings in that individual instance. No matter how small the reading of any kidney function test is, that patient is not to be deprived of the advantages of prostatectomy, provided that he shows no sign of renal insufficiency; that is, if in addition to the excretion of a good amount of urine, he shows a clean tongue, lack of any sign of interference with digestion, the presence of which would be indicated by hiccough, nausea, vomiting or soft abdominal distension. No matter how small the renal function of such a patient is, when compared with our arbitrary scale of what it ought to be, it is sufficient for him, and will be sufficient to take him through prostatectomy, provided no extra strain is thrown upon his kidneys by the operation. It has been my good fortune to do a successful prostatectomy upon at least two people whose renal function—as established by the phenolthalin test—was under 5%. There is much that suggests that the action produced upon the kidney by drainage of the over-distended bladder is purely mechanical. However this may be, following drainage the amount of urine usually goes up, the excretion of total solids increases, the retained nitrogen comes down and the excretion of salt goes up (if there has been a salt retention). In the occasional case which shows oedema, there will be a diminution of the oedema even where the intake of fluid is greatly increased. At times the primary effect of the relief of back pressure is not so favorable, but diminishes the amount of urine. This is probably due to renal congestion that follows the release of long existing pressure and may be so marked as to amount to actual anuria. Occasionally one sees bloody urine, usually of renal origin, I believe. This is more frequently seen in cases that are already infected, and represents an exacerbation of an old pyelo-nephritis, but it may occur in the cases not previously infected and while usually of renal origin may occasionally be of bladder origin. It is very important that the amount of urine excreted be carefully watched for the first three or four days after the establishment of bladder drainage and the release of the kidneys from the ill effects of back pressure, whether this is done by catheter or by suprapubic incision with prostatectomy. The first suggestion of any diminution in the amount of urine should be followed by increasing the fluid intake, for even assuming that the diminution is due to a congestion of the kidneys, the increased ingestion of fluid seems to be the method to be employed in its relief, in spite of the fact that in most conditions one might draw the conclusion that rest of the congested organs would be the most rational way to restore them to function.

When, after bladder drainage, the amount of urine goes down, water should be pushed by mouth. Where the patient's stomach will stand it, a tumbler of water per hour is desirable. Where this is not tolerated, salt solution should be given by rectum. It may be used by either the continuous method or by giving six to eight ounces every few hours. When we fail by both these means to introduce enough water to keep the amount of urine up, it is necessary to resort to subcutaneous infusion of salt solution. This can be given in much greater amounts than most people appreciate. I do not hesitate to give 750 cc. three or even four times in twenty-four hours for as long a time as this seems necessary. On one occasion, I gave a patient twenty consecutive subcutaneous infusions of 750 cc. of salt solution at eight hour intervals, before I was satisfied that the man could get on without them. This man made a very good recovery, although he was unconscious for some time and even delirious for several days. I not infrequently see this patient on the street. The only contra-indication that I see to the use of salt solution is the appearance of oedema. This will ordinarily appear first in the scrotum and with its appearance I diminish the use of salt solution.

The principal drugs which I use to stimulate urinary excretion are caffeine sodium benzoate in grain doses every few hours, given by mouth or subcutaneously. This is supplemented by digitalis, if the cardiac action is not satisfactory. Getting these patients up, either in bed or, more often, out of bed, seems to be a good stimulant to their general well-being and in this way acts as a stimulant to their renal function. I rarely allow patients of this type, particularly if they seem in poor condition, to remain quietly in bed. The same procedure for stimulating renal excretion is applicable both following draining of the bladder as a preliminary to prostatectomy and also following the operation for the removal of the prostate. If, however, the two-stage operation is done and the kidneys have been gotten into a condition of equilibrium following the drainage step, it will be unusual to have a diminution of function following the enucleation, unless ether is used as an anaesthetic. In order that one may know the amount of renal excretion, our most important and earliest indication of impending danger, it is essential that constant irrigation of the bladder following either cystostomy or prostatectomy be given up. I have for a number of years protested against this, because of my feeling that this procedure does an incalculable amount of harm, in that it prevents an accurate knowledge of the amount that the kidneys are excreting and the early adoption of adequate measures to stimulate renal action in cases where this begins to flag.

There is no arbitrary time to be placed on how long one should drain patients with urinary back pressure, before proceeding to the prostatectomy. One keeps up the drainage until the renal function is satisfactorily re-established or until the patient has made as much progress as it seems probable that he can. Where it is wise to carry out this preliminary drainage, it is seldom desirable to do the second stage of the operation under a week's time, and usually a delay of ten days or two weeks is better. I have allowed as long a time as nine weeks to elapse between the draining of the bladder and the enucleation of the prostate. However, the time which elapses should depend entirely on the patient's condition. When the patient is passing a good amount of urine, when there are no signs of indigestion, the tongue is clean, the abdomen not distended, one may assume that the patient's renal function has become re-established.

I should like to call attention here to the importance of soft abdominal distension as a sign of renal insufficiency. I believe it is an important sign of toxemia, due to faulty renal action, and has less general recognition than it should have.

Naturally one will depend somewhat upon the sort of anaesthesia he is to use for the enucleation of the prostate as to how long an interval he allows to elapse between the establishment of drainage and the enucleation or between the first and second stages of operation. If one is to use gas and oxygen, and more particularly if one is to use spinal anaesthesia, it is admissable to operate earlier than if one is to use ether anaesthesia, which irritates the kidneys to so much greater an extent. The use of ether causes a certain amount of renal congestion that is undesirable under any circumstances; when we have kidneys impaired by severe suppuration or greatly embarrassed by back pressure, the use of ether may produce a very severe degree of renal injury, sufficient, at times, even to cause anuria and death. It should be used only when the kidneys are in good condition.

My own feeling is strongly in favor of the use of spinal anaesthesia as the routine anaesthetic to employ in enucleation of the prostate. It is especially indicated in the cases that have a narrow margin of safety and when one must avoid anything that can possibly injure the renal function. It meets its clearest indication, perhaps, in the suprapubic cases. It has certain limitations when used in the perineal cases, in that it is undesirable to place the patient in the exaggerated lithotomy position for some little time following the injection. I have always waited fifteen or twenty minutes. This is due to the fact that this tilting of the patient may produce a higher anaesthesia than is safe, due to the gravitation upward of the novocaine, in case the pelvis is raised, before such time as the solution has been absorbed. Not only does spinal anaesthesia allow us to enucleate the prostate without adding any injury to the renal function, but it also diminishes the shock, and the lower blood pressure diminishes the tendency to hemorrhage. Its chief asset, however, is its lack of injurious action upon the kidneys.

It cannot be denied that spinal anaesthesia has its drawbacks, the deathly pallor which one occasionally sees, the toxic condition accompanied by vomiting and almost complete disappearance of the pulse is very trying. These, fortunately, are not very frequent accompaniments. The post-operative headache, which unfortunately, is not very rare, is a great annoyance. The rather smaller doses that we have been using of late and the less forcible injection seem to have lessened the number of toxic cases and diminished the uncomfortable features attending its use. From a personal experience of about one hundred prostatectomies done under spinal anaesthesia without fatality, I am convinced of the very great advantage of this form of anaesthesia in prostatic work. While the stormy cases frighten me and are extremely trying, I prefer this to the greater danger, amounting sometimes even to euthanasia, that general anaesthesia by ether produces in many prostatics. My experience with gas-oxygen anaesthesia has been very limited. My feeling is that its danger is probably fully as great as that of spinal anaesthesia, perhaps even greater. It has seemed to me that the tendency to bleed, following enucleation under gas-oxygen anaesthesia, is decidedly greater than it is under spinal anaesthesia, but I am unqualified through lack of familiarity with gas-oxygen to compare it with spinal anaesthesia.

I recognize the fact that the cardiac condition is a considerable danger in some of these cases that undergo prostatectomy; that the general inroad made by advanced years is also a heavy handicap, though I have done a successful prostatectomy on a man of ninety; that the trauma of operation may at times be severe and attended with marked shock; the bleeding at time of operation may occasionally jeopardize life, as may also the secondary hemorrhages that are now and then seen. These hemorrhages may, however, usually be controlled by packing the bladder by one of the several methods that have been advocated for this purpose. Sometimes a transfusion of blood is necessary when either the amount of blood lost has been very great or we have a deffciency in coagulability. It has been my experience, however, that the sum of all these dangers is not so great as the danger that arises from the lack of adequate renal preparation in the patients that are to undergo prostatectomy; that this is the important element in the treatment of these cases: the recognition of which diminishes greatly its mortality.

---

**Analysis of 133 Fractures of Spine.** J. B. Hartwell, Boston, Boston M. & S. Jour., July 12, reports on a series at the Mass. Gen. Hospital. 7 occurred in females, 126 in males. No cases were observed under 10 but the age distribution was fairly even. 83 were due to falls from a height, 25 to "jack-knife" injuries including 11 by weights, 6 by low bridges, 5 by diving, 1 each by cave-in, football and coasting injuries. 8 were due to collision with trains and 1 with automobile, 7 to direct blows, 2 to alighting from moving cars, in 7 no history was obtained. The fractures involved the cervical vertebrae in 48 cases, 7th cervical and 1st dorsal in 1, dorsal in 50, 12th dorsal and 1st lumbar in 1, lumbar in 33. The 49 cervical fracture cases involved 76 vertebrae, by lesion of two or more in a case. These included none of the first, 1 of the 2d, 7 of the 3d, 14 of the 4th, 29 of the 5th, 19 of the 6th, 6 of the 7th. The 51 dorsal cases involved 66 vertebrae, the number of each in order being as follows: 1, 3, 7, 5, 1, 2, 4, 6, 3, 9, 8, 17. The 34 lumbar cases involved 40 vertebrae, the number of each in order being: 20, 8, 1, 3, 4 and 4 unspecified. In 41 cases, other complications were noted, totaling (by combination in certain cases) 64 lesions. These included 12 fractures of the skull, 16 of ribs, with puncture of lung in 4 cases, 25 other fractures of bones, sternum, pelvic and limbs, and various visceral lesions, extensive lacerations, etc.

---

**Rollier's Method in the Treatment of Pott's Disease.** J. W. Flynn, Prescott, Ariz., S. W. Med., Oct. 1917, quotes Rollier's statistics of 198 cases: 171 completely cured, 18 improved, 5 stationary, 4 died. He pleads for the application of this method in the U. S. and considers Arizona especially available as the sun-baths can be given at all seasons and there are few sunless days. All altitudes can be had, from that of Rollier's institution down.

